# Tunnel electroresistance through organic ferroelectrics

**DOI:** 10.1038/ncomms11502

**Published:** 2016-05-04

**Authors:** B. B. Tian, J. L. Wang, S. Fusil, Y. Liu, X. L. Zhao, S. Sun, H. Shen, T. Lin, J. L. Sun, C. G. Duan, M. Bibes, A. Barthélémy, B. Dkhil, V. Garcia, X. J. Meng, J. H. Chu

**Affiliations:** 1 National Laboratory for Infrared Physics, Shanghai Institute of Technical Physics, Chinese Academy of Sciences, Shanghai 200083, China; 2 University of Chinese Academy of Sciences, Beijing 100049, China; 3 Laboratoire Structures, Propriétés et Modélisation des Solides, CentraleSupélec, CNRS-UMR8580, Université Paris-Saclay, Châtenay-Malabry 92295, France; 4 Unité Mixte de Physique, CNRS, Thales, Univ. Paris-Sud, Université Paris-Saclay, Palaiseau 91767, France; 5 Key Laboratory of Polar Materials and Devices, Ministry of Education, East China Normal University, Shanghai 200241, China

## Abstract

Organic electronics is emerging for large-area applications such as photovoltaic cells, rollable displays or electronic paper. Its future development and integration will require a simple, low-power organic memory, that can be written, erased and readout electrically. Here we demonstrate a non-volatile memory in which the ferroelectric polarisation state of an organic tunnel barrier encodes the stored information and sets the readout tunnel current. We use high-sensitivity piezoresponse force microscopy to show that films as thin as one or two layers of ferroelectric poly(vinylidene fluoride) remain switchable with low voltages. Submicron junctions based on these films display tunnel electroresistance reaching 1,000% at room temperature that is driven by ferroelectric switching and explained by electrostatic effects in a direct tunnelling regime. Our findings provide a path to develop low-cost, large-scale arrays of organic ferroelectric tunnel junctions on silicon or flexible substrates.

## Abstract (web summary)

Ferroelectric organic materials can be used for tunnel barriers in memory devices as a cheaper and eco-friendly replacement of their inorganic counterparts. Here, Tian *et al*. use poly(vinylidene fluoride) with 1–2 layer thickness to achieve giant tunnel electroresistance of 1,000% at room temperature.

## Graphical abstract

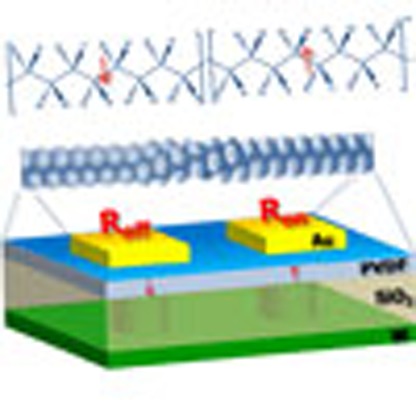

In emerging ferroelectric tunnel junctions (FTJs), switching the polarisation of an ultrathin ferroelectric barrier sandwiched between two electrodes modulates the junction resistance, giving rise to giant tunnel electroresistance (TER)^1^,^2^. This simple non-destructive resistive readout of the ferroelectric information^3^ could alleviate scalability issues encountered with the destructive capacitive readout in conventional ferroelectric random access memories^4^. In addition, FTJs open a new route for the nanoscale control of the spintronic response when using ferromagnetic metals as electrodes^5^,^6^,^7^,^8^. Although experimental evidence suggests that TER is undoubtedly associated with ferroelectric polarisation switching^3^,^9^,^10^,^11^, there are still some fundamental issues to be addressed^2^. Indeed, large TER in oxide-based tunnel junctions cannot be interpreted by simple electrostatic models involving partial screening of polarisation charges at the interfaces^12^,^13^, but necessitates complex descriptions involving interfacial dielectric layers^14^ or doped-semiconducting layer^15^. Moreover, almost all the experimentally reported FTJs are using ferroelectric barriers made of oxide perovskite thin films^2^. To maintain ferroelectricity in nanometre-thick oxide films, sophisticated experimental approaches such as strain engineering^16^ and careful control of epitaxial growth are generally required^17^, which inevitably results in a complex fabrication process, not mentioning the limitation of large-area production of such inorganic FTJs.

*En route* towards more processable FTJs, organic ferroelectric materials^18^ have also been considered as tunnel barriers^19^,^20^. Organic ferroelectrics could open up the practical applications of FTJs to silicon technology, large-area applications^21^ or flexible electronics^22^. In addition, organic FTJs may also exhibit different electronic transport properties from their inorganic counterparts because of the weak Van der Waals interfacial bonding. Recently, organic FTJs were reported using vinylidene fluoride (VDF) oligomer as ferroelectric barrier^23^. While the results are encouraging with a TER of ∼500% using these oligomers, the homogeneity of the current is questionable considering the roughness (4 nm r.m.s. for a 7.2-nm film) and large size (0.3 mm diameter) of the junctions^23^. Moreover, the electronic transport mechanism at play is still unidentified. Alternatively, poly(vinylidene fluoride) (PVDF) homopolymer and its copolymers with trifluoroethylene (P(VDF-TrFE)) are robust organic ferroelectrics. These copolymers are promising candidates for TER since their ferroelectric character persists down to nanometre thicknesses^24^,^25^: conductance switching was observed at the local scale using scanning probe microscopy across 1-nm-thick P(VDF-TrFE) films^26^ and in capacitors based on 10-nm-thick P(VDF-TrFE) films^27^. However, solid-state FTJs based on ultrathin PVDF-based films have not yet been reported and the fundamental physics of TER in organic FTJs remains to be explored.

Here, we report on the observation of a robust room-temperature TER in organic FTJs using ultrathin PVDF films (1 and 2 layers (Ls) thick, 2.2- and 4.4-nm thick, respectively). We unambiguously show that the ferroelectric switchability is sustained down to 1 layer, and reveal that charge transport across both mono- and bi-layers PVDF-based FTJs proceeds by a direct quantum-mechanical tunnelling transport. We also demonstrate a stable TER reaching 1,000%. These results are interpreted by simple electrostatic models involving consistent physical parameters for both thicknesses.

## Results

### Ferroelectricity in ultrathin films of PVDF

**Figure 1:**
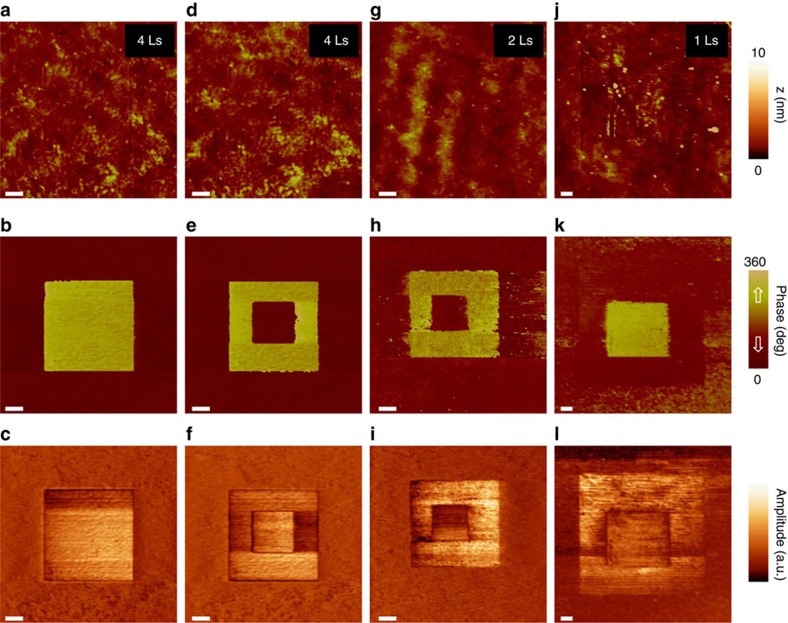
Ferroelectricity in ultrathin thin films of PVDF. (**a–c**) Topography, PFM phase and amplitude images, respectively, after switching the polarisation upward in the central 5 × 5 μm^2^ area of a 4Ls-thick PVDF film. (**d–f**) Topography, PFM phase and amplitude images, respectively, of the same area after switching back the polarisation downward in the central 2.5 × 2.5 μm^2^ area. (**g–i**) Topography, PFM phase and amplitude images, respectively, after sequentially switching the polarisation upward in the central 5 × 5 μm^2^ area and switching the polarisation downward in the central 2 × 2 μm^2^ area of a 2Ls-thick PVDF film. (**j–l**) Topography, PFM phase and amplitude images, respectively, after sequentially switching the polarisation downward in the central 10 × 10 μm^2^ area and switching the polarisation upward in the central 5 × 5 μm^2^ area of a 1Ls-thick PVDF film. Scale bar, 1 μm.

In this study, pure PVDF ultrathin films are deposited layer-by-layer by the Langmuir–Blodgett (LB) technique onto silicon wafers, using gold as a bottom electrode (see the ‘Methods' section, Supplementary Fig. 1). A major advantage of PVDF-based polymers is the ability to deposit them as thin film at room temperature on any material^28^. Figure 1 shows the out-of-plane piezoresponse force microscopy (PFM) images of ferroelectric domains written on PVDF films of 4Ls, 2Ls and 1L. In Fig. 1b, a 5 × 5 μm^2^ square with upward polarisation is defined by scanning the grounded atomic force microscopy (AFM) tip with a positive voltage applied to the bottom electrode. A clear 180° phase shift is observed relative to the pristine PVDF background with a homogeneous downward polarisation. This ferroelectric domain pattern is supported by the PFM amplitude image (Fig. 1c) that only drops at the domain wall. This square domain with upward polarisation can be switched back to its original state by scanning a 2 × 2 μm^2^ area with negative voltage (Fig. 1e,f). The topography of the film is not damaged during the writing process (Fig. 1a,d). This clear signature of ferroelectricity in 4Ls PVDF films is in line with polarisation versus field measurements demonstrated previously^25^. The bright (dark) PFM phase signal for polarisation upward (downward) is strictly opposite to that observed in reference inorganic ferroelectrics (BiFeO_3_, BaTiO_3_, and so on) in agreement with the negative piezoresponse coefficient of PVDF^29^. Similar ferroelectric domain patterns can be defined on 2Ls (Fig. 1g–i) and 1L (Fig. 1j–l) of PVDF, starting by writing polarisation upward then downward or vice versa. This indicates that the ferroelectric polarisation of 1-4Ls PVDF films is switchable, making them potentially suitable as ferroelectric tunnel barriers.

### Submicron PVDF junctions show resistance switching

**Figure 2:**
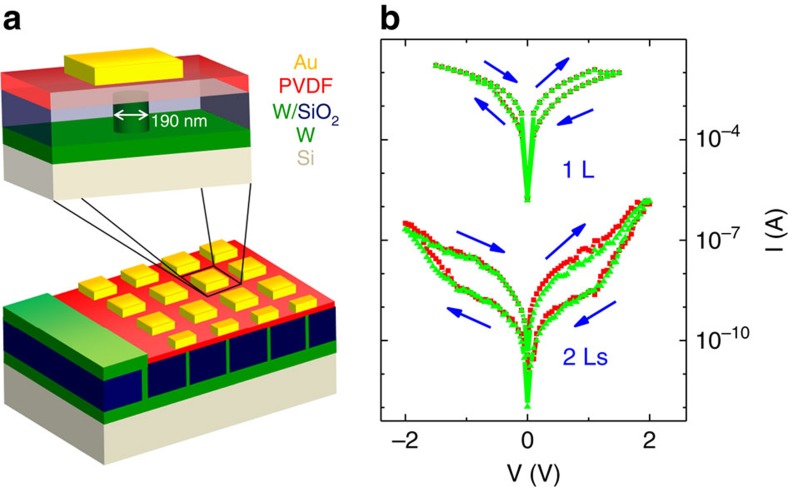
TER in submicron Au/PVDF/W tunnel junctions. (**a**) Three-dimensional sketch of the design of PVDF ferroelectric tunnel junctions. (**b**) *I*–*V* curves in the 1 and 2Ls PVDF FTJs. The arrows show the direction of the voltage sweeps. The two different data colours are obtained on two different junctions for each sample.

We design solid-state FTJs based on these ultrathin films of PVDF. As sketched in Fig. 2a, the junction size is defined by patterning the bottom electrode of W into pillars with 190 nm diameters isolated by a SiO_2_ matrix. The PVDF ultrathin films are then deposited on this template. Finally, micron-size top electrodes are deposited by Au evaporation and standard photolithography (see the ‘Methods' section). Figure 2b shows typical current–voltage (*I*–*V*) characteristics of 1L and 2Ls PVDF FTJs. A hysteresis in the current is observed for both thicknesses. For the 1L PVDF FTJ, the current switches from the OFF resistance state to the ON resistance state (that is, from small to large current flow, respectively) when sweeping the voltage on Au from +1.5 V to −1.5 V. It switches back to the ‘off' state when sweeping the voltage from −1.5 V to +1.5 V. A similar behaviour is observed for the 2Ls PVDF FTJ within a voltage window of ±2 V. The dispersion from one FTJ to another is very small as emphasized in Fig. 2b.

### Interplay between ferroelectricity and transport

**Figure 3:**
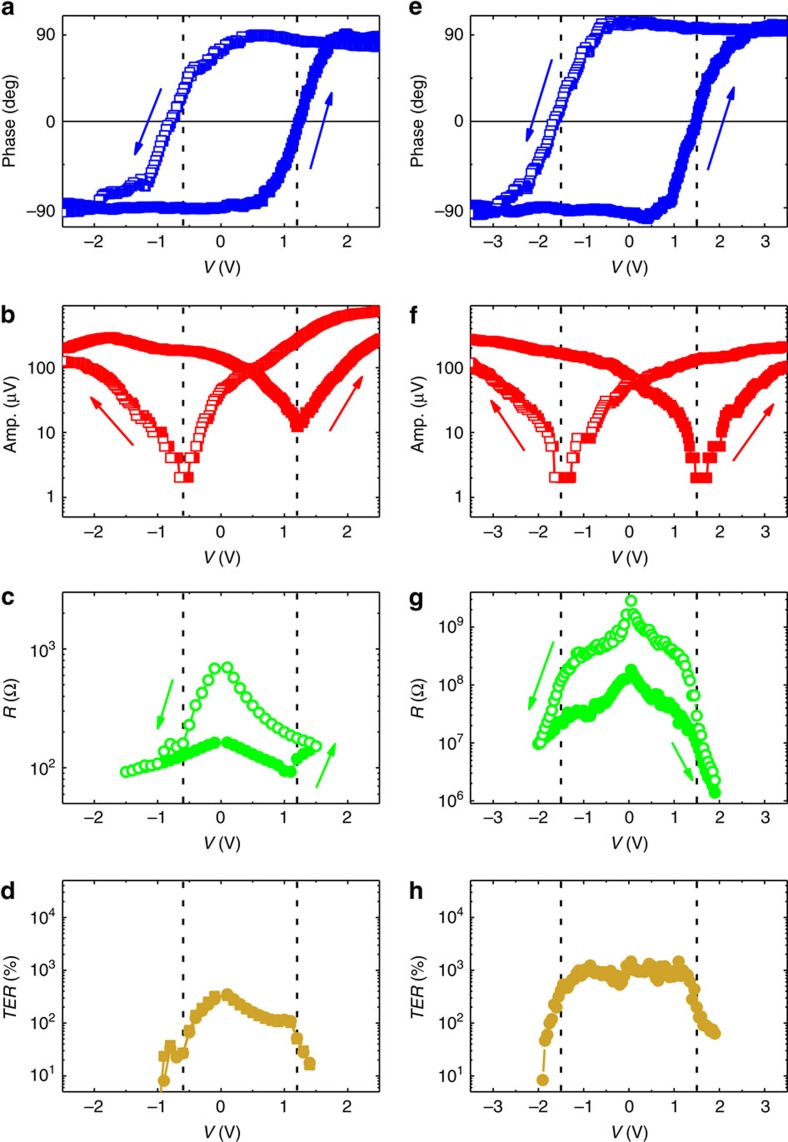
Polarisation-induced resistance switching in PVDF tunnel junctions. (**a**) Local PFM phase and (**b**) amplitude versus voltage on a 1L PVDF film deposited on Au-coated Si substrates. (**c**,**d**) Voltage dependences of the resistance and TER of a Au/PVDF (1L)/W junction, respectively. (**e**) Local PFM phase and (**f**) amplitude versus voltage on a 2Ls PVDF film deposited on Au-coated Si substrates. (**g**,**h**) Voltage dependences of the resistance and TER of a Au/PVDF (2Ls)/W tunnel junction, respectively. The vertical dashed lines show the agreement between polarisation and resistance coercive voltages.

To demonstrate that the observed resistive switching is due to polarisation reversal and not to electrochemical reactions (Supplementary Note 1), local PFM phase and amplitude loops versus voltage are performed on the PVDF films deposited on Au-coated Si substrate. The typical hysteretic PFM phase and amplitude signals of 1L PVDF (Fig. 3a,b) or 2Ls PVDF (Fig. 3e,f) films show the expected 180° phase contrasts and classical butterfly shapes for the amplitude. Fig. 3c,g show the resistance versus voltage of typical 1L PVDF and 2Ls PVDF FTJs, respectively. The coercive voltages (*V*_*C*_) for polarisation reversal (−0.6 V, +1.2 V for 1L PVDF films and ±1.5 V for 2Ls PVDF films) coincide with the voltages at which resistive switching occurs (dashed lines in Fig. 3). At negative *V*_*C*_, resistance switches from a high to a low value and the device is in the ‘on' state for polarisation pointing upward (towards the Au top electrode). Similarly at positive *V*_*C*_, the ‘off' state is reached by switching the polarisation downward (towards the W bottom electrode). This strongly suggests that the observed resistive switching is due to ferroelectric polarisation reversal in these PVDF junctions.

### Direct electron tunnelling through PDVF

Considering that the electrodes are attached to PVDF films by Van der Waals forces, changes of the electronic potential profile associated with ferroelectric polarisation switching^12^,^13^, rather than complex changes of interfacial bonds, are expected to alter the electrons transmission probability and produce TER. Possible electron transport mechanisms at play through these ultrathin films of PVDF include thermionic emission, Fowler–Nordheim and direct tunnelling^30^. Specific signatures of the different transport mechanisms can be detected by temperature-dependent transport measurements. The weak dependence of *I*–*V* curves measured between 223 and 290 K (Supplementary Fig. 3) suggests that direct tunnelling is the main transport mechanism (Supplementary Note 2). The analysis of the *I*–*V* curves at high voltage indicates a transition from direct to Fowler–Nordheim tunnelling for typical voltages of 1.3-1.5 V (Supplementary Note 3, Supplementary Fig. 4). Moreover, we estimate that direct tunnelling dominates thermionic emission in 1–2Ls-thick PVDF junctions (Supplementary Note 4, Supplementary Fig. 5). The TER (or ‘off/on' ratio) reaches ∼300% (Fig. 3d) and ∼1,000% (Fig. 3h), for 1L and 2Ls PVDF FTJ, respectively. The larger TER for thicker barriers is in line with electrostatics models based on direct tunnelling^10^,^13^.

## Discussion

**Figure 4:**
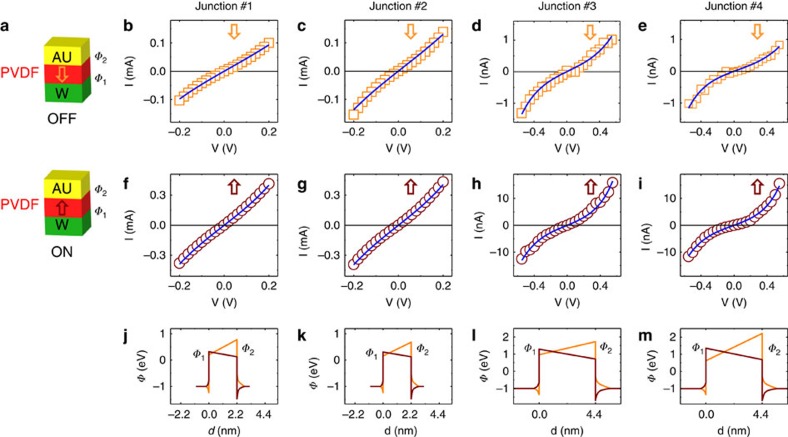
Direct quantum-mechanical tunnelling mechanism in Au/PVDF/W junctions. (**a**) Sketch of the two polarisation configurations of the junctions with the corresponding ‘on' and ‘off' resistance states. (**b**–**e**) *I*–*V* curves in the downward polarisation state for junctions #1, #2, #3 and #4. (**f**–**i**) *I*–*V* curves in the upward polarisation state for junctions #1, #2, #3 and #4. Solid lines in **b**–**i** are fits from the direct tunnelling model. (**j**–**m**) Calculated electron potential profiles in both polarisation configurations (orange and brown lines for downward and upward polarisations, respectively) for junctions #1, #2, #3 and #4. Junctions #1 and #2 are based on 1L-thick PVDF and junctions #3 and #4 for are based on 2Ls-thick PVDF.

To gain more insight into the interplay between polarisation and tunnelling, we performed *I*–*V* curves in the low-voltage range for both polarisation orientations. The results are shown for four typical junctions: two different 1L PVDF junctions (junction #1, Fig. 4b,f; junction #2, Fig. 4c,g) and two different 2Ls PVDF junctions (junction #3, Fig. 4d,h; junction #4, Fig. 4e,i). The *I–V* curves are fitted using a direct electron tunnelling model through a trapezoidal tunnel barrier^10^,^31^. We consider that the barrier has a width *d* (defined as the thickness of the PVDF film), and that the electron barrier heights are *Ф*_*1*_ at the PVDF/W interface or *Ф*_*2*_ at the Au/PVDF interface (Supplementary Note 2). Transport measurements can be accurately reproduced by the model for both polarisation orientations (solid lines in Fig. 4b–e for downward polarisation, and in Fig. 4f–i for upward polarisation) considering a constant effective mass for tunnelling electrons of m*=0.12 m_0_. The electron potential profiles resulting from the fits are displayed in Fig. 4j–m (orange and brown for downward and upward polarisation, respectively). The interfacial barrier heights vary similarly with polarisation reversal in all four junctions: when the polarisation switches from downward to upward, *Ф*_*1*_ increases while *Ф*_*2*_ decreases, and vice versa. The electron potentials decrease along the direction of the ferroelectric polarisations as expected within the framework of electrostatic models based on partial electrode screening of polarisation charges^12^,^13^. Moreover, the polarisation-induced variation of *Ф*_*1*_ is smaller than *Ф*_*2*_ (Supplementary Table 1) in agreement with the lower screening length for W (∼0.048 nm) (ref. 32) than for Au (∼0.07 nm) (ref. 8). Such variations of potential profiles with polarisation give an estimate of PVDF ferroelectric polarisation of 8–18 μC cm^−^^2^ (Supplementary Table 1) in agreement with polarisation measurements^25^ and calculations^33^. Finally, we conclude that the modulation of tunnel transmission through variations of the potential barrier shape is responsible for TER in these PVDF-based FTJs.

In summary, we demonstrate the potential of organic PVDF thin films as active ferroelectric tunnel barriers for future electronics. Pure PVDF films show room-temperature ferroelectricity down to a single layer thickness. We evidence the intimate link between polarisation switching and resistance switching in 190-nm wide solid-state nanodevices based on PVDF homopolymer, with a resulting room-temperature TER of >1,000%. The electron transport is governed by direct quantum-mechanical tunnelling with electrostatic modulations of the barrier potential profile. We believe our results will stimulate further work on organic ferroelectrics, multiferroic organic tunnel junctions^19^,^20^ and organic spintronics^34^,^35^. This will open a new route for low-cost, silicon-compatible or potentially rollable organic devices.

## Methods

### Sample preparation

PVDF films were prepared by a horizontal technology LB method. Bulk PVDF (with molecular weights of 180,275, purchased from Sigma-Aldrich) was dissolved in dimethyl sulfoxide with a concentration of 0.01 wt%. The polymer solvent was dispersed on the surface of the deionized water with a resistivity of 18.2 MΩ cm^−1^ in a KSV LB trough (Nima 611). The PVDF layers were transferred from the water-air interface with surface pressure maintained at 5 mN m^−1^ at 25 °C in an atmospheric environment.

### Thickness determination

The thickness of a ‘monolayer' PVDF film with *b*-axis orientation is ∼0.5 nm which corresponds to an ‘atomic layer'. However, 1L of PVDF film deposited by the Langmuir–Blodgget method contains 3–4 ‘atomic-layers' of PVDF^25^,^36^ or less^37^,^38^ depending on the deposition process. In this work, we performed repeated characterizations using AFM for many samples. After deposition by the LB technique, one way to measure the thickness of PVDF with an AFM is to remove the soft polymer in contact mode (with a large force) and then measure the step height of the PVDF layer in tapping mode. Examples are displayed in Supplementary Fig. 1 for many different samples. Combining these repeated AFM measurements for various numbers of PVDF layers (1, 2, 4 and 20Ls), we obtain a layer thickness of ∼2.2±0.2 nm. We note that additional conductive-AFM experiments were performed on 2Ls PVDF films to make sure that the AFM tip only remove the PVDF layer and that the Au electrode layer is preserved. The onset of uniform electric conduction during this process for an estimated thickness value of ∼4.5 nm validates the thickness determination with AFM. Such PVDF ‘layer' thickness of ∼2.2±0.2 nm fully agrees with our previous capacitance^25^ and ellipsometry^39^,^40^,^41^ measurements performed on thicker PVDF films. One example of a 20Ls PVDF sample characterization by ellipsometry is displayed in Supplementary Fig. 2. In this ellipsometry experiment, the estimated thickness is 43.457 nm for 20Ls, which corresponds to 2.2 nm per layer.

### Device fabrication

Cylinder-like W-bottom electrodes (with ∼190-nm diameter) connecting each other at the bottom were manufactured on silicon substrates using ion-beam milling combined with a standard electron-beam lithography technology (sketch in Fig. 2a). Au films with thicknesses of ∼100 nm were evaporated on top of the PVDF films. These top electrodes were fabricated under very ‘soft' deposition conditions to minimize the PVDF film damage (background vacuum of ∼10^−4^ mbar and evaporation vacuum of ∼10^−3^ mbar, distance between Au source and sample of ∼25 cm, evaporation time of <5 min). The top Au electrodes were then designed by standard photolithography and ion-beam milling.

### Physical measurements

PFM loops versus voltage measurements (Figs 3a,b,e,f) were performed at room temperature using an atomic force microscope (Bruker Multimode 8, Camarillo, CA, USA). PFM images (Fig. 1) were performed combining a lock-in amplifier (Zurich HF2LI) to track the contact resonance of the AFM tip with a multimode Nanoscope V AFM (Bruker). In both cases, commercial silicon tips coated with chromium/platinum (Budget Sensors) were used for PFM at typical contact resonance frequencies of 0.4–0.7 MHz.

*I*–*V* measurements were performed using a Keithley 6430 sub-femtoampere source meter with a remote preamplifier. Currents were collected 100 ms after the application of the external voltage. The bias voltage was applied to Au electrode both for PFM measurements and *I*–*V* measurements, while the other side (tip in PFM measurements and W electrode in *I*–*V* measurements) was grounded.

Temperature dependence of transport properties: Direct tunnelling can be experimentally distinguished from thermionic emission and Fowler–Nordheim tunnelling by its weak intrinsic temperature dependency of the resistance^11^. *I*–*V* curves between −0.3 and 0.3 V for both ‘on' and ‘off' states of junction #1 were collected at 223, 240, 260 and 290 K. The current shows low variations with temperature (Supplementary Fig. 3), which strongly suggests that direct tunnelling dominates the current flow in PVDF FTJs.

## Additional information

**How to cite this article**: Tian, B. B. *et al*. Tunnel electroresistance through organic ferroelectrics. *Nat. Commun.* 7:11502 doi: 10.1038/ncomms11502 (2016).

## Supplementary Material

Supplementary Information Supplementary Figures 1-5, Supplementary Table 1, Supplementary Notes 1-4 and Supplementary References
