# Supplementary material for: Tunnel electroresistance through organic ferroelectrics
Source: Nat Commun. 2016 May 4;7:11502. doi: 10.1038/ncomms11502 (PMC4857482; doi:10.1038/ncomms11502)
Supplement: Supplementary Information — Supplementary Figures 1-5, Supplementary Table 1, Supplementary Notes 1-4 and Supplementary References [file ncomms11502-s1.pdf]

Supplementary Figures

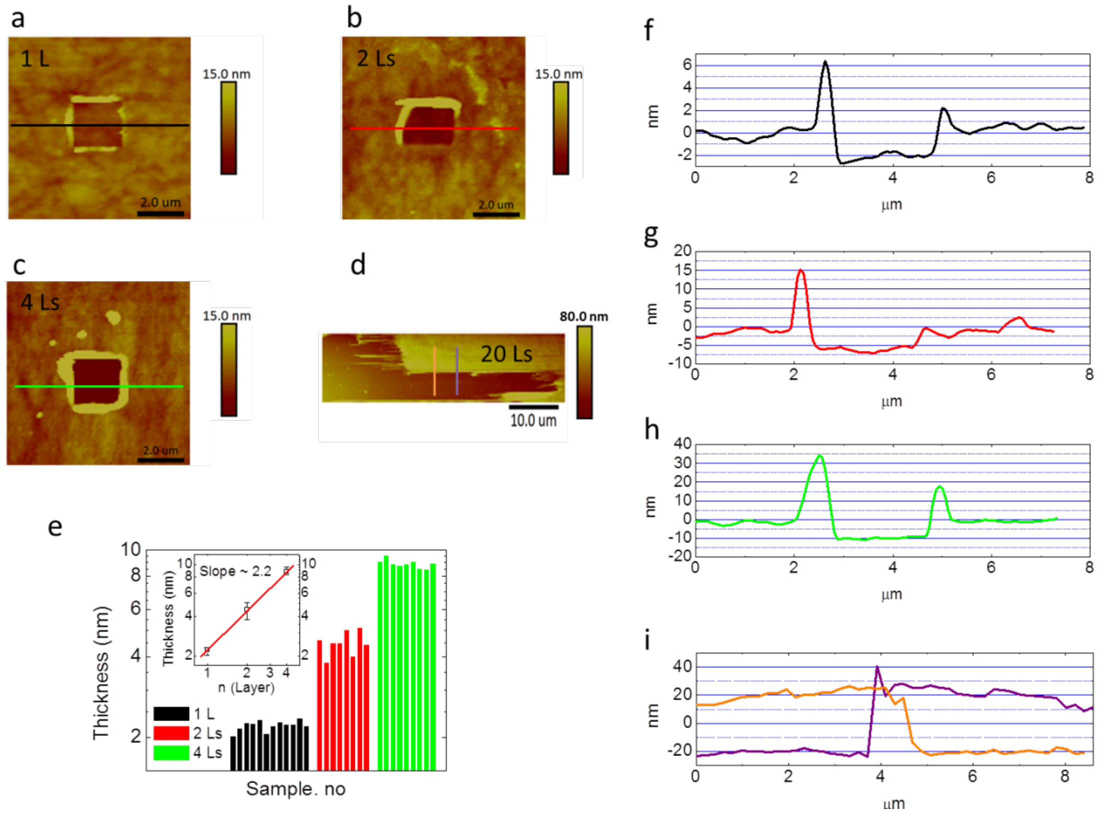

Supplementary Figure 1 | Thickness calibration of PVDF layers using atomic force microscopy. (a-d) Tapping AFM images of 1 L, 2 Ls, 4 Ls and 20 Ls PVDF films, respectively on Au-coated Si substrates. The squares in the centre (or dark region in d) are the Au-coated Si substrates revealed after removal of the film by the AFM tip (in contact mode). **(e)** Statistical data on the thickness of 1 L, 2 Ls and 4 Ls PVDF films determined by AFM as shown in (f-i). The inset in (e) shows the layer dependence of PVDF thickness. **(f-i)** Film thickness profiles along the lines in (a), (b), (c) and (d), respectively.

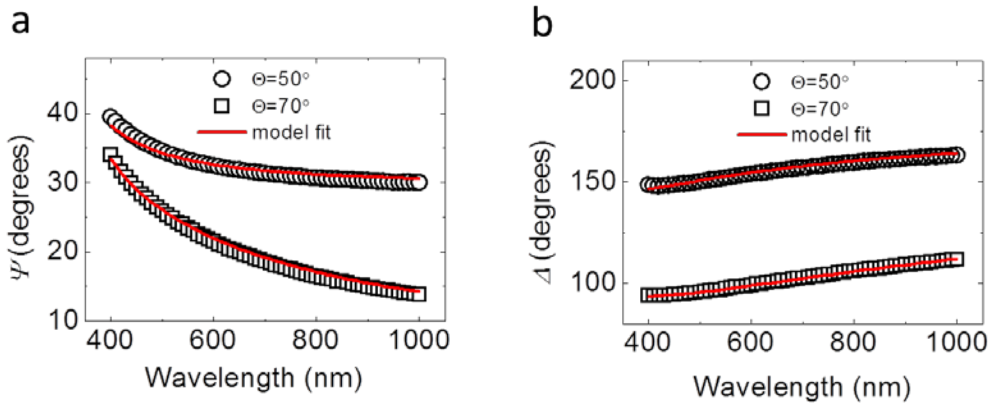

Supplementary Figure 2 | PVDF thickness using ellipsometry. (a-b) Ellipsometry measurements of Ψ and Δ, respectively from a 20 Ls PVDF film at incidence angles of Θ = 50° and 70°. The red lines show the Cauchy model fits in the 400–1000 nm spectral range obtained from the VASE multi-sample analysis.

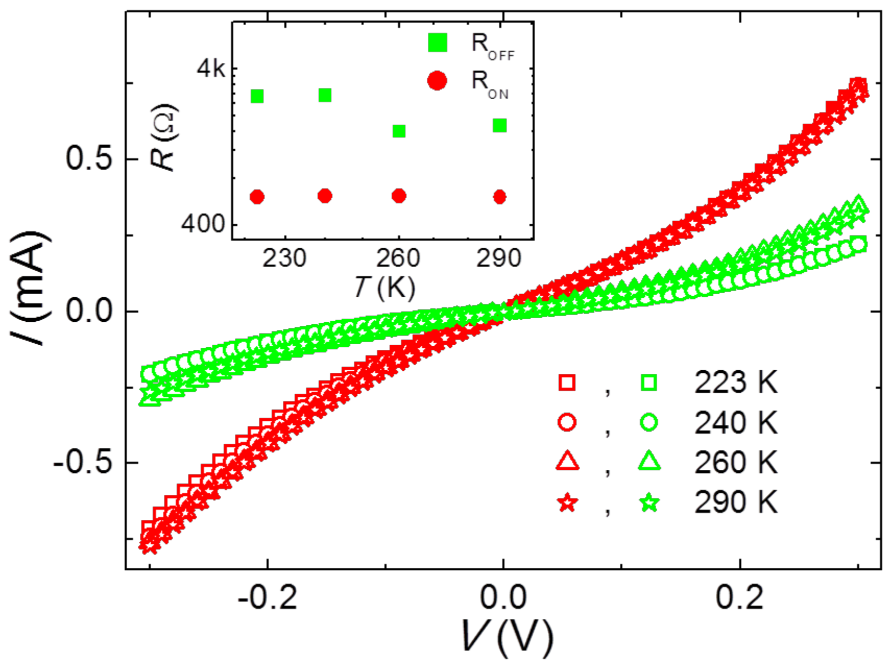

Supplementary Figure 3 | Temperature dependence of electron transport. *I-V* curves of a 2.2-nm PVDF FTJ at 223 K, 240 K, 260 K and 290 K. Inset: temperature dependence of the resistance for the ON and OFF states.

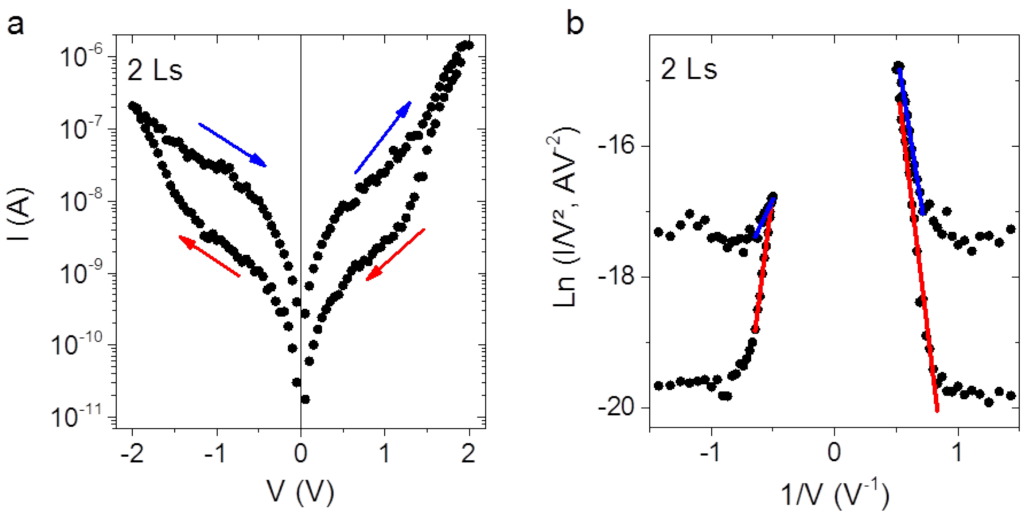

Supplementary Figure 4 | Fowler-Nordheim tunnelling transport. Analysis of the *I-V* data of a typical 4.4-nm PVDF FTJ within the FNT mechanism. **(a)** I-V curve. **(b)** $ln(I/V^2)$ versus ($1/V$) curve showing a linear dependence at high voltage. Solid lines are linear fits.

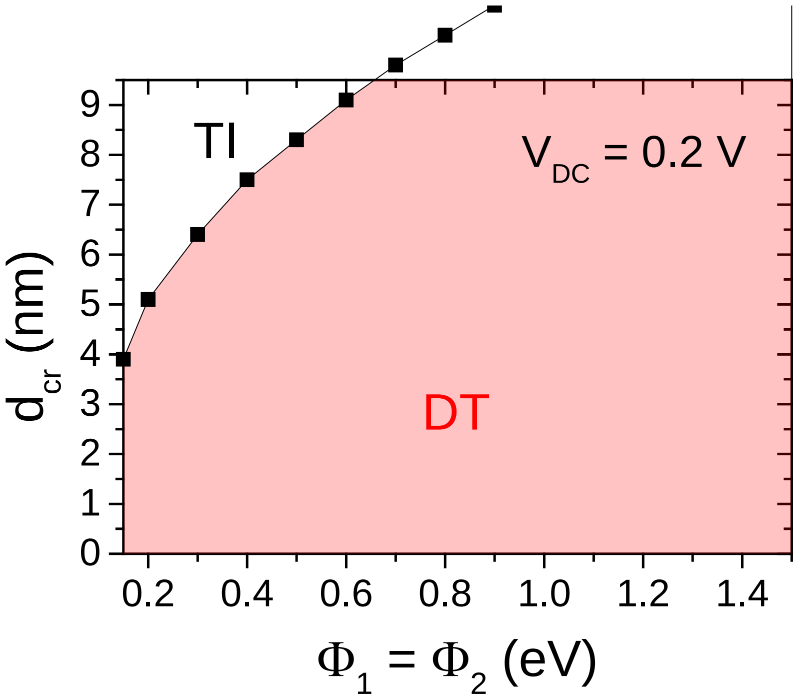

Supplementary Figure 5 | Estimated critical thickness (d_{cr}) of PVDF films below which direct tunnelling dominates thermionic injection. Several values of barrier heights (Φ_1, Φ_2) were considered. The applied voltage to electrode 2 is 0.2 V and the effective mass of carriers is $m^* = 0.12\ m_0$.

Supplementary Table

	Junction #1		Junction #2		Junction #3		Junction #4	
polarisation	down	up	down	up	down	up	down	up
thickness (nm)	2.2	2.2	2.2	2.2	4.4	4.4	4.4	4.4
Φ_1 (eV)	0.20	0.32	0.16	0.31	0.97	1.29	0.62	1.35
Φ_2 (eV)	0.78	0.13	0.68	0.13	1.72	0.72	2.21	0.69
m^* (m_0)	0.12	0.12	0.12	0.12	0.12	0.12	0.12	0.12
$\Delta\Phi_1$ (eV)	0.12		0.15		0.32		0.73	
$\Delta\Phi_2$ (eV)	-0.65		-0.55		-1.00		-1.52	
$\|\Delta\Phi\|$ (eV)	0.27		0.20		0.34		0.40	
P (μC/cm²)	14.0		10.6		15.8		18.3	

Supplementary Table 1 | Parameters of the fits of *I-V* curves across PVDF ultrathin films using the direct tunnelling model (the fits are displayed in Fig. 4 of the manuscript). Estimate of the PVDF ferroelectric polarisation using the electrostatic model.

Supplementary Note 1 | Discussion on redox mechanisms at the PVDF/W interface

An interfacial layer may exist between electrochemically active W bottom electrode and PVDF films despite our efforts to avoid such interfacial layer during the sample fabrication. For example, the substrate was protected in nitrogen atmosphere before the deposition of PVDF films, and the sample was blown by nitrogen and put upside down in the high vacuum environment (~10^{-4} mbar, ~60 °C) as soon as possible after the deposition of PVDF films. The water is believed to volatilize easily in high vacuum.

Then WO_x interfacial layers may exist and a redox-based resistive switching might be expected in Au/PVDF/WO_x/W junction if the electric field value is larger than the electric breakdown field[1]. However, no breakdown phenomenon is obtained as shown in Fig. 2 of the manuscript. Moreover, if WO_x was involved in the resistance switching of our junctions, the ON-to-OFF resistance transition would take place when a positive voltage is applied to the W bottom electrode (negative voltage on Au)[2, 3]. Indeed, the application of a positive voltage to the W bottom electrode would oxidize W at the interface (anodization of W), which would consequently lead to an increase in the junction's resistance (as WO_x is much more insulating than W). This is in contrast to our TER results with Au/PVDF/W junctions (Fig. 2b) where the ON-to-OFF transition occurs at positive voltage (i.e., negative voltage on W). Therefore, the redox-based contribution can be safely ruled out.

Supplementary Note 2 | Analysis of the transport properties within the direct tunnelling regime

Here we discuss how the *I-V* curves presented in Figs. 4b-i of the manuscript are fitted using a direct electron tunnelling model[4, 5]. We use the expression of the direct tunnelling current I_{DT} given by Gruverman *et al.*[4] in which electrons tunnel through a trapezoidal potential barrier with barrier heights Φ_1 and Φ_2:

$$I_{DT} = SC\frac{exp\left[\alpha\left\{\left(\Phi_2-\frac{eV}{2}\right)^{3/2}-\left(\Phi_1+\frac{eV}{2}\right)^{3/2}\right\}\right]}{\alpha^2\left[\sqrt{\Phi_2-\frac{eV}{2}}-\sqrt{\Phi_1+\frac{eV}{2}}\right]^2}\times\sinh\left[\frac{3eV}{4}\alpha\left\{\sqrt{\Phi_2-\frac{eV}{2}}-\sqrt{\Phi_1+\frac{eV}{2}}\right\}\right] \quad (1)$$

with $C=-\frac{32\pi em^*}{9h^3}$, $\alpha=\frac{8\pi d\sqrt{2m^*}}{3h(\Phi_1+eV-\Phi_2)}$, where S, d, h, m^* are the area of the interface, the film thickness, the Planck constant, the effective carrier mass, respectively.

The device diameter (190 nm) and the barrier thickness (d_{1L} ~ 2.2 nm, d_{2L} ~ 4.4 nm) are kept constant during the fitting procedures. Considering that the ferroelectric barriers in all four junctions are made of the same PVDF, the effective mass of tunnelling electrons (m^*) must be constant. We find that a fixed m^* = 0.12 m_0 enables to reproduce the experiments in all four junctions. Only the barrier heights on each side of the PVDF tunnel barrier (Φ_1 for PVDF/W and Φ_2 for Au/PVDF) are variables for the fittings and both are initialized at $\Phi_{1,2}$ = 1 eV. All *I-V* curves for the four junctions considered could be fitted with this classical model (solid lines in Figs. 4b-i of the manuscript). The parameters of the DT fittings are displayed in Supplementary Table 1.

As stressed in manuscript, the variations of potential profiles with polarisation can be

explained using electrostatic models based on partial electrode screening of polarisation charges. Considering the room-temperature static permittivity of ultrathin PVDF films (~6)[6], the screening length for W (~0.048 nm)[7] and Au (~0.07 nm)[8], and other parameters in Supplementary Table 1, the value of polarisation in these ultrathin PVDF films is estimated and displayed in the bottom row of Supplementary Table 1. The ferroelectric polarisation of 10-18 μC/cm² in pure PVDF films is in agreement with ab initio calculations[9] and larger than the semi-crystalline thick/bulk PVDF materials or its copolymers[10]. We note that a pessimistic value of PVDF permittivity of 2 (instead of 6) would give estimates for PVDF polarisation of 8-16 μC/cm². The physical values for the polarisation of PVDF support the overall interpretation of the results by partial screening of polarisation charges by the electrodes within a direct tunnelling model.

Supplementary Note 3 | High-voltage regime: Fowler-Nordheim tunnelling

Fowler-Nordheim tunnelling (FNT) corresponds to electrons flowing across a triangular-shaped potential barrier, which is formed by applying an electrical field *E* to a rectangular or trapezoidal barrier[11]. This mechanism dominates both DT and thermionic emission mechanisms at high applied voltages. Suppose a negative voltage is applied to metal *i*, the current is given by[11]:

$$I_{FNT} = S\frac{e^3}{8\pi h\Phi_i}\left(\frac{V}{d}\right)^2 \, exp\left[-\frac{8\pi\sqrt{2m^*}d\Phi_i^{3/2}}{3heV}\right] \quad (2)$$

Equation (2) can be rewritten as:

$$ln\left(\frac{I_{FNT}}{V^2}\right) = lnS + lnA - k\left(\frac{1}{V}\right) \quad (3)$$

where $A = \frac{e^3}{8\pi h d^2\Phi_i}$, $k = \frac{8\pi\sqrt{2m^*}d\Phi_i^{3/2}}{3he}$. At a given *d*, both *A* and *k* are constants. Then, $ln(I/V^2)$ vs. (*1/V*) is linear with a slope equal to -*k*.

We analysed the transport data displayed in Fig. 2b of the manuscript. No FNT regime could be detected in the 2.2-nm PVDF FTJ, probably because of the too low voltage window applied. In the 4.4-nm PVDF FTJ though, we could detect a change in the $ln(I/V^2)$ vs. (*1/V*) in the high voltage region, where it becomes linear with a negative slope (Supplementary Fig. 4b). We estimate the voltage threshold for the transition from DT to FNT to be in the range of 1.3-1.5 V for positive and negative voltages.

Supplementary Note 4 | Critical thickness of PVDF films for direct tunnelling

For a metal-ferroelectric-metal (MFM) junction, when the thickness of the ferroelectric films is only few nm, direct tunnelling (DT) can occur and dominate the transport current[12, 13]. For larger thicknesses of the ferroelectric layer, other transport mechanisms such as thermionic injection (TI) may dominate the electronic transport across ultrathin ferroelectric films[14]. Here, we estimate the critical thickness between DT and TI mechanisms for electron transport through PVDF.

Thermionic injection occurs when carriers overcome the potential barrier by thermal energy[15, 16]. Suppose a positive voltage is applied to metal 2, the current can be described by[14, 16]:

$$I_{TI} = SA^{*}T^{2}\, exp\left[-\frac{1}{K_B T}\left(\Phi_1 - \sqrt{\frac{e^3 E}{4\pi\varepsilon_0\varepsilon_r}}\right)\right] \quad (4)$$

where the electric field E is a superposition of the applied field $E_{ap}=V/d$ and the field due to band alignment $E_{band}=\frac{\Phi_1-\Phi_2}{ed}$. A^{*} is the effective Richardson's constant, and ε_r is the permittivity of the ferroelectric responsible for image force lowering, the value of ε_r for PVDF is about 2 ~ 3 ($\varepsilon_r = n^2$, where n is the optical dielectric constant).

We estimated the critical thickness (d_{cr}) of PVDF films for direct tunnelling using a simple model: $I_{DT} > I_{TI}$ for $d<d_{cr}$ and $I_{DT} < I_{TI}$ for $d>d_{cr}$ when the applied voltage is 0.2 V. In the calculations, $A^{*} = 1\times10^{6}$ $Am^{-2}K^{-2}$, $\varepsilon_r = 3$, $m^{*} = 0.12$ m_0, and $T = 295$ K and we vary Φ_1 and Φ_2 which are considered equal for simplicity. Supplementary Fig. 5 shows that PVDF films (2.2 and 4.4 nm) considered in the manuscript are below the critical thickness for barrier heights values obtained experimentally (Supplementary Table 1). Hence, direct tunnelling should be dominant for the films investigated in agreement with the temperature-dependent results.

Supplementary References

1. Hong, D. et al. Evolution of conduction channel and its effect on resistance switching for Au-WO_{3-x}-Au devices. *Sci. Rep.* **4** (2014).
2. Kim, S. et al. Effect of Scaling WO_x-Based RRAMs on Their Resistive Switching Characteristics. *Electron Device Letters, IEEE* **32**, 671-673 (2011).
3. Biju, K.P. et al. Resistive switching characteristics and mechanism of thermally grown WO_x thin films. *J. Appl. Phys.* **110**, 064505 (2011).
4. Gruverman, A. et al. Tunneling electroresistance effect in ferroelectric tunnel junctions at the nanoscale. *Nano Lett.* **9**, 3539-3543 (2009).
5. Brinkman, W., Dynes, R. & Rowell, J. Tunneling conductance of asymmetrical barriers. *J. Appl. Phys.* **41**, 1915-1921 (1970).
6. Mai, M., Fridkin, V., Martin, B., Leschhorn, A. & Kliem, H. The thickness dependence of the phase transition temperature in PVDF. *Physica B: Condensed Matter* **421**, 23-27 (2013).
7. Berakdar, J. & Kirschner, J. Correlation spectroscopy of surfaces, thin films, and nanostructures. (Wiley Online Library, 2004).
8. Gajek, M. et al. Tunnel junctions with multiferroic barriers. *Nat. Mater.* **6**, 296-302 (2007).
9. Nakhmanson, S., Nardelli, M.B. & Bernholc, J. Ab initio studies of polarization and piezoelectricity in vinylidene fluoride and BN-based polymers. *Phys. Rev. Lett.* **92**, 115504 (2004).
10. Furukawa, T. Ferroelectric properties of vinylidene fluoride copolymers. *Phase*

Transitions: A Multinational Journal **18**, 143-211 (1989).

11. Fowler, R.H. & Nordheim, L. in Proceedings of the Royal Society of London A: Mathematical, Physical and Engineering Sciences, Vol. 119 173-181 (The Royal Society, 1928).

12. Tsymbal, E.Y. & Kohlstedt, H. Tunneling across a ferroelectric. *Science* **313**, 181-183 (2006).

13. Kohlstedt, H., Pertsev, N., Contreras, J.R. & Waser, R. Theoretical current-voltage characteristics of ferroelectric tunnel junctions. *Phys. Rev. B* **72**, 125341 (2005).

14. Pantel, D. & Alexe, M. Electroresistance effects in ferroelectric tunnel barriers. *Phys. Rev. B* **82**, 134105 (2010).

15. Kao, K.-C. & Hwang, W. Electrical transport in solids: with particular reference to organic semiconductors. (Taylor & Francis, 1979).

16. Das, R.R., Bhattacharya, P., Perez, W., Katiyar, R.S. & Bhalla, A. Leakage current characteristics of laser-ablated $SrBi_2Nb_2O_9$ thin films. *Appl. Phys. Lett.* **81**, 880-882 (2002).
